# Characterization of acquired anemia in children by iron metabolism parameters

**DOI:** 10.1038/s41598-022-06574-0

**Published:** 2022-02-17

**Authors:** Yael Ben-David, Ariel Koren, Raul Colodner, Carina Levin

**Affiliations:** 1 Pediatric Department B, Emek Medical Center, Afula, Israel; 2 Pediatric Hematology and Research Units, Emek Medical Center, Rabin Rd, 1834111 Afula, Israel; 3 The Ruth and Baruch Rappaport School of Medicine, Technion – Israel Institute of Technology, Haifa, Israel; 4 Laboratory Department, Emek Medical Center, Afula, Israel

**Keywords:** Inflammation, Gastrointestinal diseases, Haematological diseases, Diagnostic markers

## Abstract

Inflammatory states are associated with anemia of chronic disease and acute infection. Hepcidin, a regulator of iron metabolism, is involved in iron pathophysiology during inflammation. We investigated biochemical characteristics in children with anemia from different causes. Four patient groups (n = 38; mean age: 12.44 ± 4.35 years) were studied: (1) inflammatory bowel disease (IBD, 10 patients); (2) iron deficiency anemia (IDA, 12); (3) celiac disease (CD, 8); (4) acute infection (AI, 8). Laboratory measurements were evaluated at diagnosis: blood count, serum iron, transferrin, ferritin, vitamin B_12_, folic acid, CRP, erythropoietin, hepcidin and soluble transferrin receptor (sTfR). IDA patients had the lowest Hgb (6.9 ± 1.7 g/dL), MCV (63.2 ± 7.2 fL), iron (16.8 ± 13.5 µg/dL), ferritin (4.5 ± 4.5 ng/mL) and hepcidin (3.1 ± 0.8 ng/mL) values, and the highest transferrin and sTfR values. AI patients had the highest ferritin (156.2 ± 124.5 ng/mL), CRP (144.6 ± 94 mg/L) and hepcidin (74.67 ± 12.3 ng/ml) values. Overall, hepcidin levels correlated with CRP and with ferritin (r = 0.83 and 0.85, respectively). Elucidating specific etiology-related biochemical profiles in pediatric patients with anemia from different causes using a combination of laboratory biomarkers, including hepcidin, can help physicians treat the anemia.

## Introduction

Anemia is frequently encountered in the pediatric population; the most common etiology is iron deficiency (ID). However, other conditions, such as anemia of chronic disease (ACD) and acute infection (AI), often coexist with ID and affect iron status as well, although not necessarily via iron deficiency^1,2^.

There are several commonly used biomarkers for evaluation of iron status. According to the 2020 WHO guidelines, measurement of serum ferritin assesses storage iron, while measurements of serum iron and the percentage of transferrin saturation reflect the iron supply to tissues. Serum transferrin receptor (sTfR), erythrocyte ferritin and red cell zinc protoporphyrin are indicators of the iron supply to bone marrow. The use of iron by the bone marrow can be assessed by the percentage of hypochromic red blood cells, mean corpuscular volume and reticulocyte hemoglobin content. As these biomarkers are affected by other conditions such as age, sex, disease, smoking, infection, and inflammation, it may be difficult to identify a unique indicator of iron status^3^. Diagnosis of iron deficiency anemia (IDA) relies on measurements of serum iron (SI), transferrin, transferrin saturation and ferritin in subjects with microcytic/hypochromic anemia. Hepcidin levels in those patients are low, and soluble transferrin receptors (sTfR) are high^2^.

In a recent paper concerning IDA, Russo et al.^4^ used hemoglobin level, MCV, reticulocytes, transferrin saturation and ferritin were to identify IDA patients. Increase in reticulocytes, as well as a rise in hemoglobin, were sensitive indicators for the response to iron supplementation.Anemia of inflammation, also referred to as ACD, is usually mild to moderate and develops in the context of inflammation due to decreased production of erythrocytes, and a modest reduction in erythrocyte survival^5^. The disorder, like IDA, is characterized by low SI, but it differs from IDA in that iron stores are preserved. Thus, ACD is primarily a disorder of iron distribution^5^. Inflammatory mediators such as IL-6 and IL-2 raise hepcidin levels; these, in turn, lower iron availability when there is no real iron deficiency^6^. In these patients, laboratory findings are suggestive of, but not specific for ID. They include normocytic normochromic anemia, and low SI with no concomitant increase in transferrin or sTfR. Erythropoietin (EPO) level is mildly elevated, but the response to EPO is diminished^7^. Ferritin levels might be high due to acute or chronic inflammation^6,8^. In chronic disease, there might be a combination of ACD and IDA. In practice, these markers are less helpful, and tools to distinguish between the two conditions are required^1,8^.

Anemia in celiac disease (CD) is not clearly defined. CD is considered an autoimmune condition in response to gluten antigens that results in local inflammation in the small intestine. Anemia in CD is considered IDA because of iron malabsorption, and laboratory findings are usually consistent with ID, which means low SI and ferritin, and high transferrin levels. Bel’mer et al*.*^9^ also found increased hepcidin levels in up to 20% of patients with CD and anemia, suggesting an inflammatory etiology as well.

Anemia has been well described in the course of AI, but its cause is unknown. Mean corpuscle volume (MCV), mean corpuscle hemoglobin (MCH), mean corpuscle hemoglobin concentration (MCHC) and red blood cell (RBC) distribution width (RDW) in children with AI and anemia were the same as in children without infection, and iron markers were normal^10^. Another report found that children with AI have high hepcidin and IL-2 levels, and low SI^11^.

In this study, we further define and differentiate laboratory biomarkers in four groups of children with newly diagnosed anemia due to different causes: inflammatory bowel disease (IBD), IDA, CD and anemia in AI, to better understand the pathophysiology of anemia in those patients toward providing the appropriate treatment.

## Methods

### Study population

This is a prospective, single center, cross sectional study. Pediatric patients (age range 5–20 years) who presented with anemia at Emek Medical Center, Israel, between the years 2015–2018, were enrolled. Anemia was diagnosed in all of the patients at enrollment, and was defined as Hb levels < 11.5 g/dl for ages 2–7 years, Hb level < 12.0 g/dl for ages 8–11 years, Hb level < 12.0 g/dl for females aged 12–18, Hb level < 12.5 g/dl for males aged 12–14 and Hb level < 13.0 g/dl for males aged 14–18 years^12^. Blood samples were obtained before the patients received any iron treatment and prior to initiation of any specific treatment for the assumed diagnosis. The patients were subdivided into four groups by diagnosis at enrollment: (1) IBD (representing ACD); (2) pure IDA; (3) CD; (4) AI.

### Laboratory testing

Samples were analyzed for blood count, reticulocytes, reticulocyte hemoglobin content (CHr), SI, transferrin, ferritin, vitamin B_12_, folic acid, c-reactive protein (CRP), EPO, hepcidin and sTfR. The sTfR:ferritin and sTfR:log ferritin ratios were calculated.

### Statistical analysis

Statistical analysis was performed using SPSS software. We used the Kruskal–Wallis test—a non-parametric analysis for independent samples that does not assume a normal distribution of the residuals. A significant Kruskal–Wallis test indicates that at least one sample stochastically dominates one other sample. To analyze the specific sample pairs for stochastic dominance, Dunn's test, or pairwise Mann–Whitney test without Bonferroni correction were used. Correlation with categorical parameters was performed by t-test, and *p* < 0.05 was considered significant.

### Ethics approval and consent to participate

Informed consent was obtained from the subjects' guardians. The research was approved by the Ethics Committee of Emek Medical Center, Israel (study protocol EMC-0041-15) and was consistent with the principles outlined in an internationally recognized standard for the ethical conduct of human research. All methods were performed in accordance with the relevant guidelines and regulations.

## Results

### Patient characteristics

The study included 38 patients: 10 (26%) with IBD, 12 (32%) with IDA, 8 (21%) with CD, and 8 (21%) with AI. Average age was significantly higher in the IBD group than in the other groups (*p* < 0.05). Age, gender and ethnic origin are presented in Table 1.

**Table 1:** Demographic characteristics of study population (n = 38).

	IBD n = 10	IDA n = 12	CD n = 8	AI n = 8	All n = 38
Age (years) Avg ± STD	15.1 ± 3.6	13.9 ± 3.7	10.88 ± 3.9	8.5 ± 3.3	12.44 ± 4.3 *p* < 0.05
**Gender**					
Male	3	2	3	1	9 (24%)
Female	7	10	5	7	29 (76%) NS
**Ethnicity**					
Arab	3	8	2	3	16
Jew	7	4	6	5	22

IBD, inflammatory bowel disease; IDA, iron deficiency anemia; CD, celiac disease; AI, acute infection; NS, not significant.

### Blood count characteristics and CRP as an inflammatory marker (Table 2)

**Table 2:** Laboratory parameters of the study population.

Parameter, units (reference values)	IBD Avg ± STD	IDA Avg ± STD	CD Avg ± STD	AI Avg ± STD	*P-*value
Hgb, g/dL (11–14)	11.2 ± 1.6	6.9 ± 1.7	11.3 ± 1.3	10.9 ± 0.7	< 0.001
MCV, fL (75–85)	76.53 ± 6.52	63.26 ± 7.29	73.96 ± 8.03	68.25 ± 24.87	0.004
MCH, pg (25–30)	24.75 ± 3.16	18.13 ± 3.67	23.69 ± 4.12	25.99 ± 1.43	0.001
MCHC, g/dL (32–36)	32.23 ± 1.64	28.35 ± 2.99	32.08 ± 2.34	33.71 ± 1.6	0.001
RDW, % (11.5–16)	14.6 ± 1.5	17.7 ± 1.9	14.7 ± 1.7	13 ± 1	< 0.001
Reticulocytes, % (0.5–2.5)	1.52 ± 0.16	1.35 ± 0.87	0.98 ± 0.28	0.63 ± 0.33	0.002
CHr, pg (24.5–31.8)	26.1 ± 3.7	19.8 ± 3.6	26.4 ± 4.8	24.2 ± 2.5	0.002
WBC, K/µL (4.4–11.5)	9 ± 5.3	5.8 ± 1.5	7.4 ± 1.7	12 ± 4.7	0.004
ANC, K/μL (1.5–8.5)	5.73 ± 3.39	3.03 ± 0.97	3.76 ± 1.11	9.32 ± 4.45	< 0.001
CRP, mg/L (0–5)	6.3 ± 6.2	2?? ± 2.6	1.4 ± 1.5	144.6 ± 94	< 0.001
Fe, µg/dL (40–145)	31.9 ± 30.6	16.8 ± 13.5	41.7 ± 29.1	24.4 ± 21.7	0.041
Transferrin, mg/dL (200–360)	275.7 ± 75.4	355.5 ± 22.9	336.2 ± 41.1	229.7 ± 23.7	< 0.001
Serum transferrin receptor, µg/mL (0.2–1.69)^a^	2 ± 1.1	5.7 ± 2	2.3 ± 1.3	1.4 ± 0.2	< 0.001
Ferritin, ng/mL (10–322)	18.4 ± 17	4.5 ± 4.5	9 ± 6.9	156.2 ± 124.5	< 0.001
Hepcidin, ng/mL (0.25–47.66)^a^	6.8 ± 3.6	3.1 ± 0.8	6.6 ± 5.9	74.6 ± 12.3	< 0.001
EPO, mIU/mL (4–29)	26.1 ± 20.4	311.2 ± 254.9	25 ± 21.5	15.7 ± 8.4	< 0.001
sTfRx 100:ferritin	24.3 ± 27	273.2 ± 183.5	87.3 ± 138	1.4 ± 1.2	< 0.001
sTfR:log ferritin	2.1 ± 1.7	23.4 ± 21.5	2.4 ± 2.6	0.69 ± 0.24	< 0.001

Normal laboratory values, for average age, are shown in parentheses.

IBD, inflammatory bowel disease; IDA, iron deficiency anemia; CD, celiac disease; AI, acute infection; Hgb, hemoglobin; MCV, mean corpuscular volume; MCH, mean corpuscular hemoglobin; MCHC, mean corpuscular hemoglobin concentration; RDW, red blood cell distribution width; CHr, content of Hgb in reticulocytes; WBC, white blood cells; ANC, absolute neutrophil count; EPO, erythropoietin; CRP, C reactive protein; sTfR, soluble transferrin receptor.

^a^Reference range is taken from the official ELISA kits protocols that were used, Hepcidin 25 (bioactive) HS ELISA and BioVendor Human sTfR ELISA.

Average Hgb was 9.84 ± 2.48 g/dL. Hgb was significantly lower in the IDA group than in the other groups (*p* < 0.001; Table 2). MCV, MCH and MCHC were significantly lower and RDW was significantly higher in the IDA group compared to the other groups (*p* = 0.004, 0.001, 0.001 and < 0.001, respectively; Table 2). In the AI group, percent reticulocytes was lowest, and leukocytes (WBC), absolute neutrophil count (ANC) and CRP were highest compared to the other groups. Average CRP level was higher in the IBD group than in the CD and IDA groups, but not significantly so.

### Iron indices and Hgb metabolism factors (Table 2)

SI levels were low in the whole cohort, average 27.66 ± 24.9 μg/dL. It was lowest in the IDA group (*p* = 0.041) and it was also low, albeit not significantly so, in the AI group (Table 2). CHr was significantly lower in the IDA group than in the other groups (*p* = 0.002; Table 2). Transferrin levels were highest in the IDA group and lowest in the AI group (Table 2). There was a reverse correlation between transferrin and ferritin levels (r = − 0.57, *p* < 0.05). Levels of sTfR were also significantly higher in the IDA group than in the other groups (*p* < 0.001), whereas ferritin levels were significantly lower in the IDA patients (*p* ≤ 0.01), and significantly higher in the AI group (*p* < 0.01) than in the other groups, and correlated well with CRP (r = 0.84, *p* = 0.05). Ferritin levels in the IBD and CD groups were relatively low, but higher than in the IDA group (Table 2).

We calculated the sTfRx 100:ferritin and sTfR:log ferritin ratios, previously reported as sensitive markers of iron deficiency^8,13^. Both indices were significantly higher in the IDA group than in the other groups (*p* < 0.001; Table 2). Vitamin B12 levels were within normal limits, but they were significantly lower in the IBD group compared to the other groups (*p* = 0.02). Folic acid levels were normal in all groups. EPO was significantly higher in the IDA group than in the other groups (*p* < 0.001), and the AI group had the lowest level of EPO, but the difference was not significant (Table 2).

Hepcidin levels were lowest in the IDA group and highest in the AI group (both *p* < 0.001). There was no difference in hepcidin levels between the IBD and CD groups. There was a positive correlation between hepcidin and CRP, and hepcidin and ferritin levels (r = 0.83, *p* < 0.05, r = 0.85, *p* = 0.05, respectively; Table 2).

## Discussion

In this prospective study, we characterized the biochemical profiles of common types of acquired anemia in children, with an emphasis on the less routinely evaluated factors: hepcidin, EPO and sTfR. This is the first work published in the English-language literature that compares these four groups with anemia (IBD, IDA, CD and AI) for factors additional to routine iron status analysis.

Complete blood count (CBC) and iron status in the different causes of anemia are well established, and most of our findings matched previous reports^2,5^. CHr is considered a more sensitive marker for iron deficiency and changes earlier than ferritin or transferrin saturation^13^. The use of CHr as a diagnostic biomarker for ID in pediatric IBD patients was evaluated by Syed et al.^14^. but was not found to be more sensitive than other commonly used markers. Our results showed significantly lower CHr in the IDA group compared to the other groups, consistent with prior reports. Interestingly, in all groups, the CHr value was below the threshold level for iron deficiency (28 pg)^13^. This suggests that functional ID is present in both chronic disease and AI. In the following, we limit our discussion to hepcidin, EPO and sTfR.

### Hepcidin

Hepcidin is a liver-derived peptide that was discovered in 2001 and has a key role in the regulation of systemic iron homeostasis. It is known as a master regulator of iron metabolism and is regulated by iron, inflammation, and erythropoiesis. This peptide inhibits the intestinal absorption of iron, inhibits the recycling of iron derived from catabolism of senescent RBC in macrophages, and prevents mobilization of liver iron stores^15^. Hepcidin production is increased by excess iron and inflammation, and suppressed by both ID and increased erythropoiesis^16^. Inflammatory cytokines, such as IL-6, upregulate hepcidin expression by activating the IL-6R/JAK2/STAT3 pathway. High hepcidin levels induce iron retention in macrophages, high serum ferritin levels and iron-restricted erythropoiesis, all features of anemia of inflammation. Several new molecules that regulate hepcidin expression have been recently discovered^18–20^. They include protein 6 (BMP6)/SMAD1/5/8 pathway, which is influenced by high iron levels and stimulates hepcidin expression in hepatic cells; and TMPRSS6, which is required for EPO-mediated hepcidin suppression in mice. In inflammation, proinflammatory cytokines, especially IL-6, induce hepcidin production, which reduces iron absorption and iron release from macrophages, subsequently reducing iron bioavailability^6,17^.

Manipulation of the hepcidin–ferroportin axis is the most logical experimental approach to managing iron disorders. The rationale is to use hepcidin agonists for iron-overload disorders caused by inappropriate low hepcidin levels, and hepcidin antagonists to release sequestered iron in IDA and ACD^17^.

In our cohort, as expected, we found that hepcidin levels were significantly higher in AI, and lower, but still elevated, in ACD. We observed a similar degree of mild elevation of hepcidin levels in both IBD and CD and a low level of hepcidin in IDA. Hepcidin levels correlated well with CRP and ferritin (r = 0.85), demonstrating that hepcidin activity is proportional to the degree of inflammation. The moderate hepcidin increase in patients with IBD can be explained by the chronic nature of the disease. However, this mild increase seemed to diminish the response to anemia, with EPO, as well as sTfR levels remaining low, despite low SI.

Martinelli et al.^18^ demonstrated similar results in patients with IBD: high hepcidin levels in patients with active IBD compared to patients with CD and healthy controls. In that study, the hepcidin levels correlated with CRP and erythrocyte sedimentation rate. Karaskova et al.^6^ demonstrated that patients newly diagnosed with Crohn's disease had higher levels of hepcidin than patients with ulcerative colitis, which correlated well with CRP. The authors deduced that in ulcerative colitis, the anemia is mainly IDA rather than ACD. In contrast, Paköz et al.^21^ did not show a good correlation between hepcidin and CRP levels in patients with IBD, and suggested that hepcidin is not as good a marker for inflammation.

As expected, we found the lowest levels of hepcidin in patients with IDA. Erythroferrone, which is produced by erythroblasts, acts on hepatocytes to suppress hepcidin production and thereby increase iron absorption and stored iron mobilization. In this group, hepcidin levels were so distinctively low that, in combination with other iron status and CBC measurements, hepcidin could be used to better differentiate between IDA and ACD, as suggested previously^1,21^.

In the group of patients with CD, hepcidin levels were similar to those seen in patients with IBD. This suggests that anemia in patients with CD shares elements of both iron deficiency and chronic inflammation. These findings conflict with Martinelli et al.^18^ who showed significantly higher hepcidin levels in ACD compared to CD and controls, but are supported by Bel’mer et al.^9^ who showed that 20% of patients with CD demonstrate an ACD pattern with an increase in hepcidin and IL-2 levels.

In the group of children with AI and anemia, hepcidins levels were much higher compared to the other groups, including IBD, with inverse correlations to SI, transferrin and sTfR. The role of hepcidin as an antimicrobial protein has been recently shown by Stefanova et al.^23^ Our results imply that the role of hepcidin in AI is more prominent than in chronic inflammation and might cause acute anemia and prevent iron bioavailability. Moran-Lev et al.^10^ reported high levels of hepcidin that correlated with high levels of IL-2 in patients with AI. In the current study, hepcidin levels correlated well with CRP and ferritin, which may reflect high cytokine levels. Since hepcidin has a meaningful antibacterial effect in infection, it remains to be seen whether targeting it to prevent anemia in the acutely ill child is beneficial or detrimental.

### EPO and sTfR

**Figure 1:**
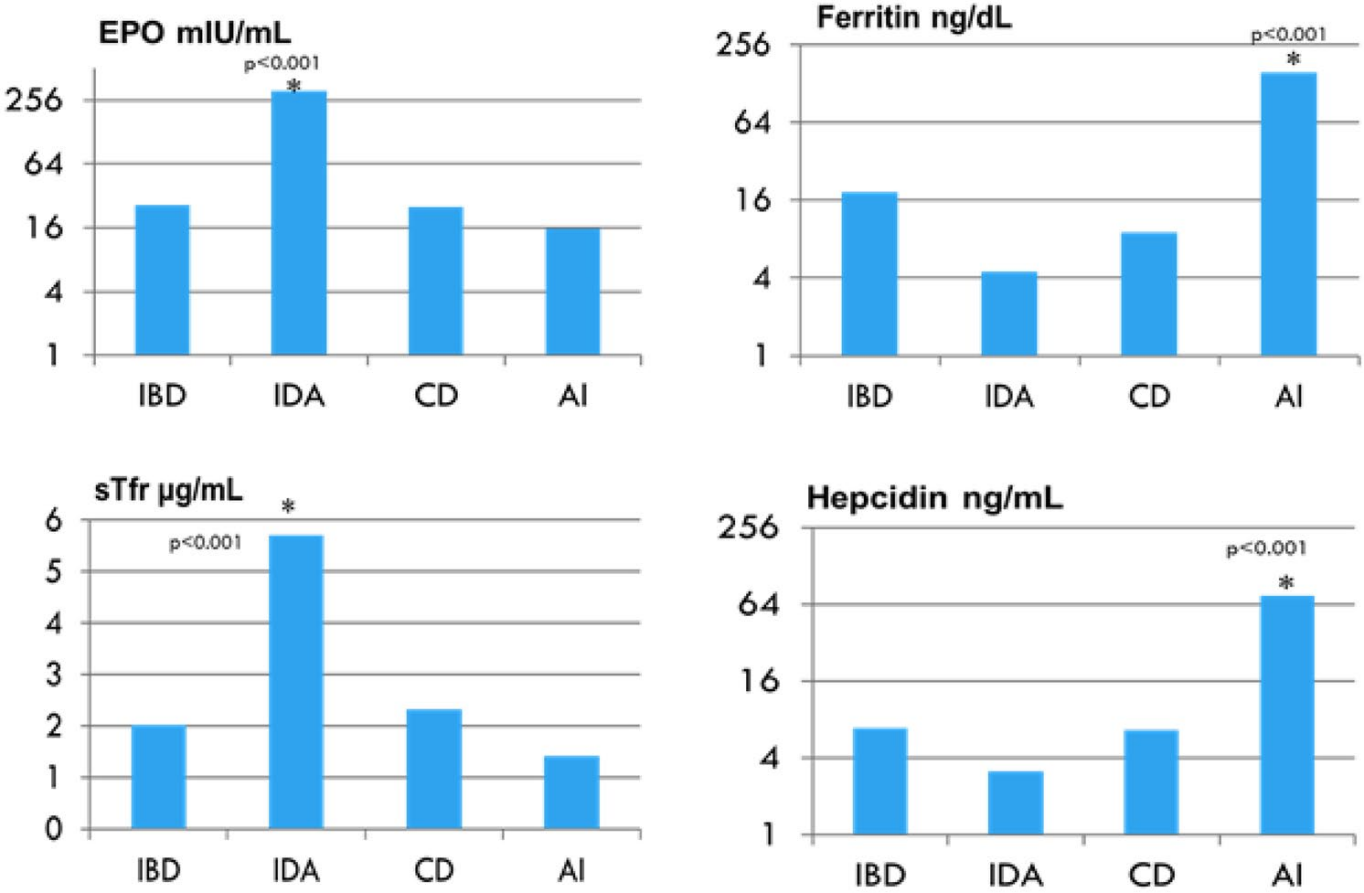
Comparing the main biochemical parameters in four groups of anemia in children. IBD, inflammatory bowel disease; IDA, iron deficiency anemia; CD, celiac disease; AI, acute infection; EPO, erythropoietin; sTfR, soluble transferrin receptor.

The EPO and sTfR levels were significantly higher in patients with IDA and lower in the group with AI (Fig. 1). This is consistent with previous reports showing that sTfR and EPO are mainly affected by iron status and not inflammation, and therefore might be more specific to IDA. Arezes et al.^24^ and Mirciov et al.^20^ showed that EPO suppresses hepcidin production through erythroferrone synthesis. Our findings of significantly high levels of EPO and decreased levels of hepcidin in the IDA patients, with high levels of transferrin and sTfR, support this.

Hershko^1^ discussed the need for a better diagnostic marker for IDA. Along with hepcidin, transferrin and sTfR could be useful for differentiating IDA from ACD. Mahajan et al.^22^ concluded that high sTfR levels (≥ 3 µg/mL) characterize patients with ACD and IDA, whereas normal sTfR levels (< 3 µg/mL) are seen in patients with pure ACD. In our cohort, the sTfR level was < 3 µg/mL in all groups except the IDA group.

Oustamanolakis et al. reported that the sTfR:ferritin (sTfR:log ferritin) index can be used to identify ID in patients with IBD. A ratio of < 1 was attributed to ACD and excluded ID and a ratio of > 2 was attributed to IDA or a combination pathology^2,8,13^. sTfRx 100:ferritin and sTfR:log ferritin ratios were both significantly higher in IDA compared to the other groups. The sTfR:log ferritin ratio was lowest in patients with AI (0.69), and higher, but still low (< 3), in the IBD and CD groups, all significantly different from the IDA group. This ratio is indicative of a combination of chronic inflammation and ID as the mechanism for anemia in patients with IBD and CD; those ratios can then be used to establish a diagnostic biochemical profile for ACD. In the group of patients with CD, the levels of sTfR and EPO were low and similar to those in the IBD group. CRP levels were lower in patients with CD compared to those with IBD. However, in patients with CD, ferritin levels were low and transferrin levels were high, as seen in IDA. This suggests a combination of pathogeneses in CD:ID and inflammation, supported by the high hepcidin levels in these patients. Interestingly, sTfR:ferritin and sTfR:log ferritin values in our CD patients were low and similar to the results seen in the IBD group, supporting a more prominent inflammatory component as the cause of the anemia.

Children with anemia in AI had very low levels of SI, transferrin and sTfR, with inverse correlations to serum hepcidin. sTfR:log ferritin was < 1, suggesting a "purer" inflammatory pathophysiology. The EPO and reticulocyte values were reduced, demonstrating suppression of erythropoiesis in AI.

## Limitations

A major limitation of our study is the small cohort of patients studied, and a small number of patients in each group. Despite this, our results showed clear statistical significance, probably because of well-established inclusion criteria for each group. The groups are not balanced in the respect to gender and age, so the average biomarkers levels may not represent the general population. However, it seems that for different etiologies for anemia in our region there is a difference in the patients characteristics (i.e. in the IDA group most of our patients were female adolescents, probably due to menstrual bleeding, and in the AI group most were younger children where gender difference is less critical regarding iron status). We believe that the results do represent the average patient characteristics in our region. This may be different concerning other world regions and therefore similar studies in other countries are recommended and until those studies will be performed, the results of this study may not be universally generalizable.

## Conclusions

In this study, we characterized the specific iron-status biochemical profiles in children with anemia related to different etiologies. The combination of routine laboratory biomarkers with specific analyses, including hepcidin, EPO and sTfR, can help physicians elucidate the pathophysiology, diagnose, and treat children with anemia caused by different etiologies.

Our findings emphasize the combined pathophysiology in ACD, the elucidation of which requires more than one marker. In CD, our results suggest that inflammation is predominant, similar to patients with IBD, combined with pure iron deficiency; this is in contrast to the currently accepted explanation. The biomarker profile in patients with AI correlated well with the increased inflammatory response.

Further research is required to establish the practicality of including these analyses in the clinical setting. The issue of treating or preventing ACD and anemia of AI warrants further investigation, to determine whether such an approach is beneficial or detrimental to the patients. In other words, do we really need to give iron treatment in ACD? Or will the anemia resolve itself when we achieve control of the disease, and thus decrease hepcidin levels, enabling mobilization of the sufficient stores of iron? The "spontaneous" improvement of anemia of AI suggests that this may also be the case in ACD.

## Data Availability

The datasets generated during and/or analyzed during the current study are not publicly available due to the privacy policy concerning private information of patients but are available from the corresponding author on reasonable request.

## Abbreviations

IBD: Inflammatory bowel disease
IDA: Iron deficiency anemia
CD: Celiac disease
AI: Acute infection
Hgb: Hemoglobin
MCV: Mean corpuscular volume
MCH: Mean corpuscular hemoglobin
MCHC: Mean corpuscular hemoglobin concentration
RDW: Red blood cell distribution width
CHr: Content of Hgb in reticulocytes
WBC: White blood cells
ANC: Absolute neutrophil count
EPO: Erythropoietin
CRP: C reactive protein
sTfR: Serum transferrin receptor
NS: Not significant
